# Lightweight Polyethylene/Hexagonal Boron Nitride Hybrid Thermal Conductor Fabricated by Melt Compounding Plus Salt Leaching

**DOI:** 10.3390/polym14050852

**Published:** 2022-02-22

**Authors:** He-Jie Pi, Xiao-Xiao Liu, Jian-Yu Liao, Yue-Yun Zhou, Cong Meng

**Affiliations:** 1 College of Urban and Environment Sciences, Hunan University of Technology, 88 Taishan Road, Zhuzhou 412007, China; pihejie@163.com (H.-J.P.); zyenn@sina.com (Y.-Y.Z.); mengcong629@163.com (C.M.); 2 Hunan Provincial Key Laboratory of Comprehensive Utilization of Agricultural and Animal Husbandry Waste Resources, Hunan University of Technology, 88 Taishan Road, Zhuzhou 412007, China; 3 Hunan Key Laboratory of Water Safety Discharge in Urban and Its Resource Utilization, Hunan University of Technology, 88 Taishan Road, Zhuzhou 412007, China; 4 School of Advanced Manufacturing Technology, Guangdong Mechanical & Electrical Polytechnic, Guangzhou 510550, China

**Keywords:** porous thermal conductor, hexagonal boron nitride, specific thermal conductivity, open-cellular, polymer composites

## Abstract

Application of porous polymeric materials is severely limited by their ultralow thermal conductivities. Herein, by promoting the formation of thermal conduction pathways, we fabricated open-cellular structured polyethylene/hexagonal boron nitride hybrid thermal conductors via melt compounding plus salt leaching. The structural analyses indicate that the inclusion of hBN can enhance the open-cell level of resultant materials. X-ray diffractions confirm the high in-plane alignments of hBN in each sample. Consequently, the test results evidence the superior thermal conductivities of our samples, and the thermal conductivities of each sample are characterized as functions of hBN loadings. Ultimately, our advanced porous thermal conductor with a low hBN loading of 3.1 vol% exhibits a high specific thermal conductivity of 0.75 (W/mk)/(g/cm^3^), which is 82.9% higher than virgin PE and far higher than bulk PE/hBN composites. Our work also intends to reveal the architectural advantages of open-cellular, as compared with the close-one, in fabricating porous materials with highly interconnected fillers.

## 1. Introduction

Porous polymeric material (PPM), also known as polymer foam, is one of the most important polymeric materials that own cellular architecture [1]. Benefit from the high contents of pores composing gas inside, PPMs show outstanding characteristics of lightweight, large surface area and reinforced specific mechanical properties [2]. Currently, PPMs have received considerable attention since they can promote the development of structuring technologies for the next generation of construction, automotive, aerospace, medical applications, communication device, and triboelectric nanogenerator, etc. [3,4,5,6].

It is widely accepted that the large volume fraction of gas inside PPMs brings these characteristics. However, due to the ultralow thermal conductivity (λ) of gas, which is around 0.02–0.03 W/mk as characterized by Montgomery et al. [7], PPMs are highly thermal insulate [8,9,10]. These thermal insulative PPMs cannot satisfy demands for lightweight thermal conductor of current personal electronics. In recent years, investigators have been trying to include thermal conductivity enhancers into PPMs to tailor their thermal conduction abilities and produce porous thermal conductors (PTCs). Among all fillers been used, hexagonal boron nitride (hBN) attracts lots of attention due to its high λ and electric insulation nature [11]. For instance, Chan et al. [12] used hBN to enhance the thermal conductivities of linear low-density polyethylene (LDPE) through foaming, and demonstrated the effect of foaming parameters on the thermal conductivities of resultant samples. Leung et al. [13] reported the effects of foam volume expansion and cell size on the λ value of resultant polyethylene (PE)/hBN foams. Besides, there are literatures dealing with the theoretical work of filler included in PTCs [9,14]. Although notable experimental and theoretical progress have been made in this area, the obtained thermal conduction abilities of PTCs are still far away from meeting industrial applications needs. Specifically, most of the reported foams show even lower λ values than the bulk ones.

As been well investigated previously [15,16,17,18], the heat transfer in polymers is dominated by phonon transportation. For hBN reinforced polymeric materials, the heat transfer behavior can be altered by the phonon transitions in and between hBN and polymer matrix, thus, the formation of hBNs’ network is essential not only as pathways for phonons transportation, but also avoid their scatterings in the polymer/gas interfaces. Previous studies [12,13] have proved that the processing parameter and PTCs’ composition can influence the dispersion of hBN and promote the formation of thermal conduction pathways. By taking a deep investigation into the previous work, it is easy to see that all these reported hBN included PTCs are close-cellular architectures. If we imagine the PTCs as a bi-phase composite with major phase of gas and minor phase of polymer, it is reasonable to consider such hBN included close-cellular PTC as a sea-island structural or matrix-dispersion structural composite, where the polymer/hBN and gas serves as the “sea” and “island”, respectively. There is a reasonable inference that the fillers in a co-continuous structure are easier to be interconnected rather than in a sea-island structure, which has been widely proved in electric conductive polymeric composites [19,20,21,22,23]. Following such concept, we assume that the hBN filled PTCs with open-cellular structure could exhibit pronounced enhancement on thermal conductivity. Currently, to the best of our knowledge, there is no report in this field.

Thus, for the purpose of understanding how cellular architecture specifically affect the hBN interconnectivity as well as develop heat transfer in PTCs, for the first time we fabricated PE/hBN hybrid PTCs with open-cellular structure. A facile melt compounding plus salt leaching technology was employed for constructing such open-cellular architectures in the composites. Limited with the small range of observation, the scanning electron microscopy (SEM) is considered unable to provide enough information of the as-obtain porous structure. Thus, in this work, the open-cellular structure was primarily analyzed by calculating the porosities and connectivities. To obtain more knowledge of the structure, X-ray diffraction (XRD) was applied to identify the crystalline information of as-prepared materials. 

## 2. Materials and Methods

### 2.1. Materials

PE (CAS: 9002-88-4) powders (grade code 7000F) were purchased from Mitsui Chemicals Co. (Tokyo, Japan) with melt flow index of 0.04 g/10 min and density of 0.95 g/cm^3^. Pristine hBN (CAS: 10043-11-5) with average diameter of 20 µm (see Figure S1) and purity ≥ 98.5% (according to the manufacture information) was obtained from Suzhou Nutpool Materials Technology Co. (Suzhou, China). The salt used in this work is commercially available NaCl (CAS: 7647-14-5, purity ≥ 97.2%) and bought from a local market. All materials were used as received.

### 2.2. Sample Fabrication

**Figure 1:**
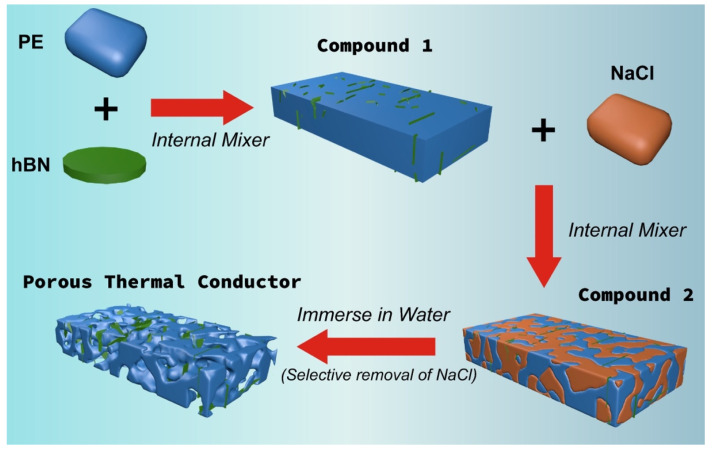
Schematic illustration of the fabrication procedure of PE/hBN hybrid PTCs.

In this work, PTCs were fabricated using the melt compounding plus salt leaching technology, and the working process was illustrated in Figure 1. Before processing, the PE, hBN and salt were dried in vacuum oven at 60 °C for more than 6 h to remove the residual moisture. To guarantee the selective localization of hBN in PE phase during melt compounding, a two-step process was employed. In the first step, the pre-blended PE and hBN with specific compositions (see Table 1) were fed into a Brabender internal mixer to prepare compound **1** (C1). After pre-compounding, C1 was cooled down in air, and then blended with salt to obtain the final compound (C2) via the same internal mixer. For both step 1 and step 2, the melt compounding conditions were set with rotation speed at 60 rpm, temperature at 195 °C and compounding time of 6 min. After melt compounding, the products were compression molded into 1-mm thick sheets at 195 °C with pressure of 15 MPa for 240 s. Afterwards, the sheets were immersed into warm water (60 °C) for 48 h to remove salts. Consequently, the porous samples were gently collected and dried for further characterizations.

In application, porous polymer composites show significant advantage about the low apparent volume fraction of the additional filler, which is due to the large content of pores. In this work, the apparent volume fraction ($V_{BN}$) of hBN in PTC can be calculated by using the equation as below:

$$V_{BN}=\frac{\rho_{1}\times W_{BN}}{\rho_{BN}}$$

where $\rho_{1}$ is the density of sample before salt leaching (compound **1**), $\rho_{BN}$ is the density of hBN we used, $W_{BN}$ is the weight fraction of hBN depends on the specific composition. The $V_{BN}$ of each sample is calculated and included in Table 1.

### 2.3. Characterization

#### 2.3.1. Scanning Electron Microscopy (SEM)

The structures of cryo-fractured surfaces of PTCs with various hBN loadings were analyzed by SEM imaging (S-3700N, Hitachi, Japan) on fractured samples. The specimens were soaked in liquid nitrogen for 30-min to be frozen, and then were broken to reveal internal structures. All samples were covered with Au before subjected to SEM imaging, and the applied voltage is 5 kV with a working distance of ~11 mm at room temperature (20 °C).

#### 2.3.2. Porous Structure Analysis

In addition to use SEM for morphology evaluation, the porous structures of as-prepared PTCs were also analyzed using two parameters: porosity (P) and connectivity (C). Herein, the porosities and connectivities of PTCs were calculated as follows:

$$P=\frac{\rho_{1}-\rho_{2}}{\rho_{1}}\times 100\%=\frac{m_{1}-m_{2}}{m_{1}}\times 100\%$$

$$C=\frac{m_{1}-m_{2}}{m_{salt}}$$

where *ρ*_1_ and $\rho_{2}$ are the apparent densities of the samples before and after salt leaching, respectively, and can be measured by density meter. $m_{1}$ and $m_{2}$ are the weights of the sample before and after salt leaching, respectively. $m_{salt}$ is the theoretical weigh of salt included in the sample and can be calculated as follows:

$$m_{salt}=m_{1}\times W_{salt}$$

where the weight fraction of salt ($W_{salt}$) can be found in Table 1.

#### 2.3.3. X-ray Diffraction (XRD)

The crystalline information of polymer, hBN and PTCs were all characterized by a X’pert3 Powder X-ray diffractometer (PANalytical; Cu, λ = 0.15418 nm, 40 kV, 40 mA) with a range of 2θ from 10~70° at room temperature. The scanning step of 0.01° was measured at 1 °/min.

#### 2.3.4. Thermal Conductivity Evaluation

Hot disk method is a transient evaluation of thermal conductivity based on a transient pulse heating technique (see Figure S2). In this work, TPS2500 Hot Disk instrument (AB Corporation, Stockholm, Sweden) with the anisotropic mode was used to performance the thermal conductivity measurement of the samples in both in-plane and through-plane directions. During measurement, a thin plane, which is typically made by Kapton and called electrically insulated resistive element, is placed between two test samples as temperature sensor and heat source. The sensor supplied a 0.03 W heat pulse to the sample for 20 s. The resistance increase *vs* time during heating was recorded with an electrical current pulse, and the thermal conductivity can be deduced.

## 3. Results and Discussion

**Figure 2:**
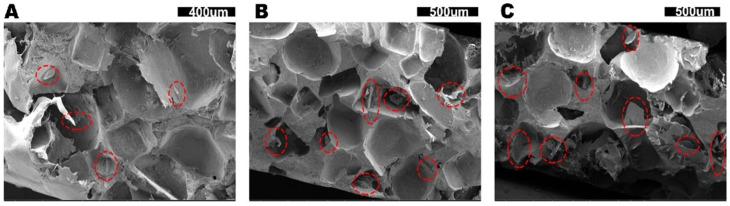
The SEM images of our novel PTCs with various hBN loadings, (**A**) 5 wt%, (**B**) 10 wt% and (**C**) 15 wt%. The hBN sheets are marked by red cycles.

Photographs of as-prepared PTCs are shown in Figure S3. From the pictures, we can see all samples show white color, which is due to the presence of hBN. In addition, several dark points are observed on the sample surface that can be assigned to the pores. The images of freeze fracture PTCs samples under SEM are shown in Figure 2. From these images, we can see lots of cubic or round pores exist in all samples, which is due to the removal of NaCl. Besides, several hBN sheets can be seen from the images (marked by red cycles) indicating that the hBN sheets can cross the PE boundaries between the pores. Moreover, no free-standing hBN sheets are observed, which confirms that the primary location of hBN is in the PE matrix. Taking a deeper investigation into these samples, it is clear to see that for sample with increased hBN loading, there are more interconnected pores. However, limited with the small observation range of SEM, the result is unable to provide quantitative information about our open-cell structure. Additional techniques are required for a comprehensive understanding of the structure evolutions.

**Figure 3:**
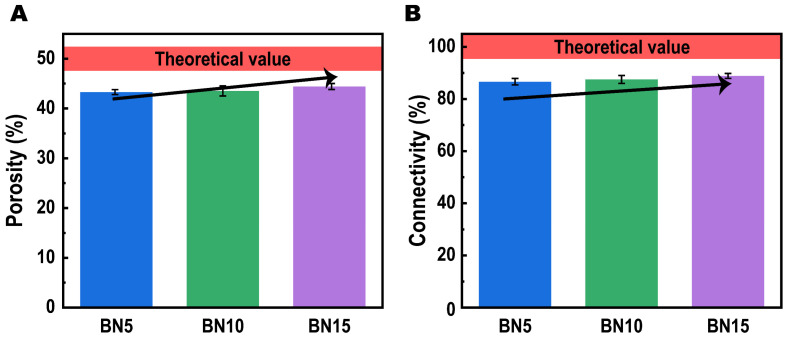
The calculated porosities (**A**) and connectivities (**B**) of each PTCs we prepared.

Therefore, to analyze the porous structures of PTCs we prepared, the porosities and connectivities of these samples were also calculated. Typically, the porosity and connectivity of a porous material represent the void fraction and open cell level, respectively [24,25]. Thus, for our salt leaching generated porous samples, the theoretical porosity depends on the loading of the salt and is calculated as 50% in this work, while the theoretical connectivity should be 100%. However, both the experimental porosities and connectivities of our samples present obvious negative deviations from the theoretical values as shown in Figure 3A,B. This phenomenon results from a frequent issue in current salt leaching technology that the salt is not completely penetrated [26]. Even after hot water leaching for 72 h, there are still some salts left in the porous material due to inconsecutive dispersion. Considering this issue, we applied a simple calculation and found the weigh fractions of residual NaCl for BN5, BN10 and BN15 are 6.7 wt%, 6.4 wt% and 5.6 wt%, respectively. As been investigated by Kutcherov et al. [27], the inclusion of NaCl in PE at low fraction (<15 vol%) has little effect on the thermal conductivity at room temperature. Thus, we assume that for each sample, the effect of residual NaCl on thermal conductivities is negligible.

In order to quantitatively analyze the effect of hBN on the porous structure of as-prepared PTCs, we made a further study of the porosities and connectivities, then find their clear increasing trend with more hBN inclusion. The porosity rises from 43.3% for 5 wt% hBN to 44.4% for 15 wt% hBN, while the connectivity varies from 86.6% for BN5 to 88.8% for BN15. The results suggest that the hBN can promote the interconnection of salt due to its larger size than NaCl.

**Figure 4:**
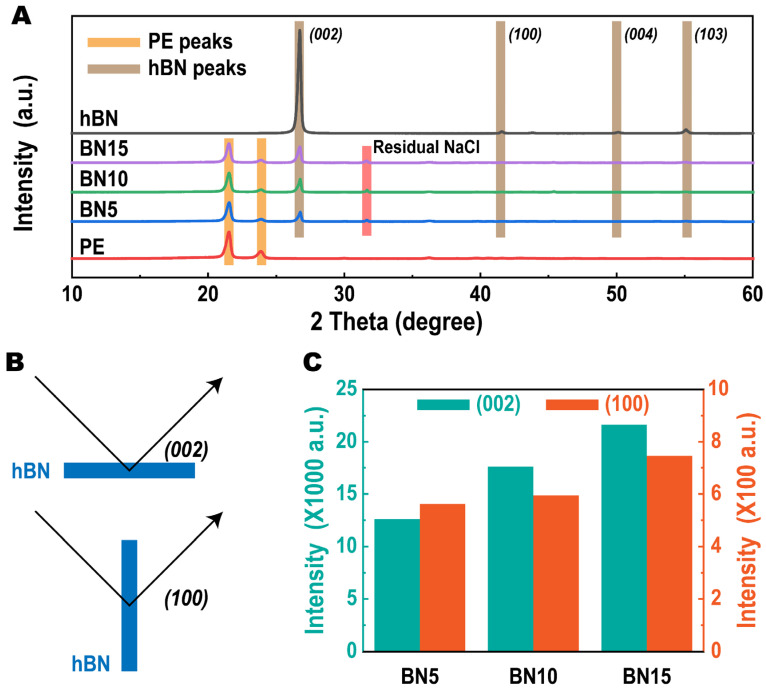
(**A**) XRD patterns of porous PE/hBN hybrid thermal conductors with various hBN loadings. The XRD patterns of virgin PE and hBN are also included for reference. The diffractions belong to PE, hBN and residual NaCl are marked by orange, brown and red squares, respectively. (**B**) Illustrating the effect of the orientation of hBN on the XRD pattern: horizontally oriented hBN is responsible for (002) peak (top) and vertically oriented hBN is related to the (100) peak (bottom). (**C**) Intensity variations of (002) and (100) peaks of hBN in each sample.

Figure 4 shows the XRD patterns of samples with various hBN loadings. The peak intensities in each XRD patterns were collected as shown in Table S1. Virgin PE and hBN we used in this work are also included for reference. From the XRD pattern of virgin PE, there are two peaks locating at ~22° and ~24°, respectively, which present the (110) and (020) planes of PE crystal. The XRD pattern of hBN shows four characteristic peaks at ~26°, ~42°, ~50° and ~55°, respectively, that can be assigned to the (002), (100), (004) and (103) planes of the crystal. The XRD pattern of the hBN is consistent with previous reports that confirms its hexagonal structure [28].

For the porous PE/hBN blends, all characteristic peaks of PE and hBN can be observed in the XRD patterns. An additional peak at ~33° can be noticed regardless of the hBN loadings in the composites. According to literatures [29,30], this peak belongs to the residual NaCl crystals after salt leaching process, which is consistent with the previous porosities and conductivities calculation results. Besides, it is noted that in the XRD patterns of BN5, BN10 and BN15, the (004) and (103) peaks seem to be disappeared. Such disappearance of peaks in Figure 4A is believed due to the low magnification of the figure.

Taking a deep investigation into the XRD patterns of samples with different hBN loadings, it can be seen that the intensity of (002) peak increases with more hBN inclusion. Such an increase should be due to more hBN layers’ response to the X-ray. Moreover, as shown in Figure 4B, the (002) and (100) diffractions are generated from hBNs that place horizontally and vertically to the X-ray, respectively. Thus, the ratio (δ) of (002) peak intensity to (100) peak intensity is normally used as a sign of the hBNs’ alignment [31,32,33], which can be calculated as follows:

$$\delta \left(\%\right)=\frac{I_{\left(100\right)}}{I_{\left(100\right)}+I_{\left(002\right)}}\times 100$$

where $I_{\left(100\right)}$ and $I_{\left(002\right)}$ are the intensities of (100) peak and (002) peak, respectively, that can be obtained from the XRD patterns. For clarity, the intensities of (002) and (100) peaks are collected and plotted in Figure 4C. From these columns, it is clear to see that both $I_{\left(002\right)}$ and $I_{\left(100\right)}$ increase with more hBN loading, which is due to more hBN layers’ response to the X-ray. The calculated δ values show no obvious variations with different hBN loadings, indicating the fraction of hBN has insignificant effect on its alignment. Besides, the δ of all samples locate at ~3.3%, which suggests a strong alignment of hBN. It is believed that such phenomenon is induced by the hot compression technique during specimens preparation [34].

**Figure 5:**
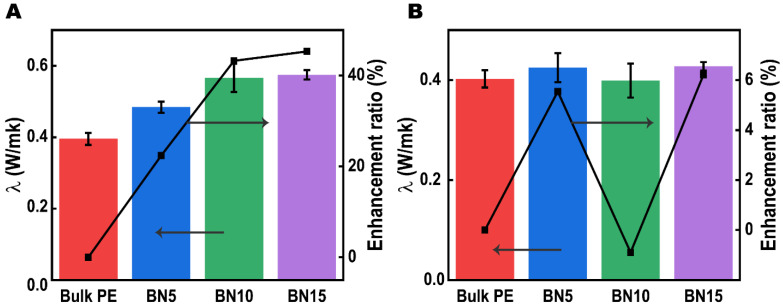
In-plane (**A**) and through-plane (**B**) thermal conductivities of porous PE/hBN hybrid thermal conductors with various hBN loadings.

Next, based on the above investigations of the highly interconnected open-cellular structure, we examined the thermal conductive performance of our PTCs, while the bulk PE is also tested for reference. Given to the anisotropic 2D structure of hBN, the examinations were conducted in an anisotropic mode with in-plane λ ($\lambda_{∥}$) and through-plane λ ($\lambda_{⊥}$) monitored, and the results are plotted in Figure 5A,B, respectively. Among all tested samples, the bulk PE displayed an isotropic thermal conductive behavior with $\lambda_{∥}$ and $\lambda_{⊥}$ of 0.39 W/mk and 0.40 W/mk, respectively, which is in agreement with previous literatures [35,36,37]. Such low λ value also represents the thermal insulation nature of PE. Typically, PPMs can be considered as a bi-component material that consists of low-λ polymers and ultralow-λ gas [8,38], which further develops the thermal insulation of materials and makes it difficult in fabricating low-density and high-λ thermal conductive polymeric foams [8]. However, for our open-cellular structural PTCs, enhanced λ is observed in the in-plane direction (see Figure 5A). Practically, the $\lambda_{∥}$ increased from 0.48 W/mk for BN5 to 0.56 W/mk for sample BN10, which is up to 46.2% higher than the virgin PE. The upward trend becomes gentle when including more hBN, since BN15 shows barely increased $\lambda_{∥}$ of 0.57 W/mk. It is well accepted that, for hBN included polymeric materials, the connection of hBN plays a key role in enhancing the thermal conductivity of the composites. Specifically, Alvarez-Lainez et al. [39] investigated that the formation of open-cellular architecture in polymer foams can facilitate the thermal convection rather than the conventional close-cellular structure. Herein, the enhanced thermal conductivities of our samples also suggests that the formation of open-cellular structure can promote the formation of hBN connection paths.

Unlike the in-plane thermal conduction reinforcement induced by inclusion of hBN, the through-plane λ does not show any obvious variations (see Figure 5B). The $\lambda_{⊥}$ of each sample locates around 0.4 W/mk regardless of the hBN loadings. Furthermore, we calculate the anisotropic index (AI) of each sample by $\mathrm{AI}=\lambda_{∥}/\lambda_{⊥}$. As result, the AI values are 1.13, 1.40 and 1.33 for sample BN5, BN10 and BN15, respectively, which indicates an anisotropic thermal conductivity enhancement. Because of the natural anisotropic thermal conductivity of hBN, the anisotropy in thermal conductivities is believed due to the high alignment of hBN as confirmed by XRD analysis (Figure 4).

**Figure 6:**
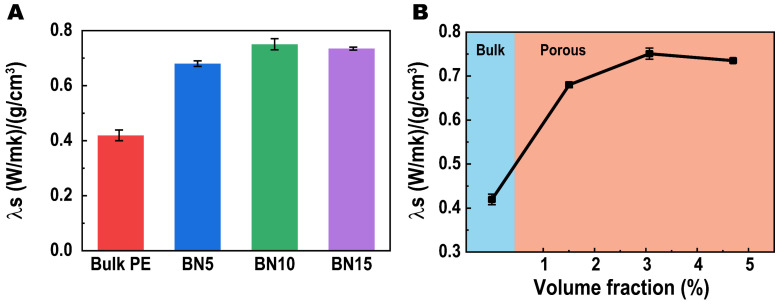
(**A**) Specific thermal conductivities ($\lambda_{S}$) of our samples, and (**B**) the specific thermal conductivity ($\lambda_{S}$) *vs* apparent volume fraction of hBN of each PTC.

As we mentioned above, for applications including small-size communication devices, the lightweight feature of materials is essential. Thus, it leads to a desire for developing outstanding thermal conductive materials with relatively low density. Such performance can be quantitatively evaluated by the specific thermal conductivity ($\lambda_{S}$) of material as $\lambda_{S}=\lambda /\rho$ [40,41]. Herein, we calculated the $\lambda_{S}$ of our TCPs as shown in Figure 6A. Comparing the $\lambda_{S}$ with $\lambda_{∥}$ values given above, $\lambda_{S}$ shows significantly higher value in each sample including the bulk PE, which is due to their low densities (<1 g/cm^3^) as marked in Table 1. Normally, for bulk polymer/hBN blends, their densities are proportional to the hBN loading because of the relative high density of hBN (2.29 g/cm^3^ theatrically). If we calculate it theoretically, the PE/hBN composites should exhibit lower $\lambda_{S}$ than $\lambda_{∥}$ when the hBN loading exceeds 4 wt%. However, the hBN inclusion in our composites with considerable λ enhancement far surpasses this critical value, which leads to an insignificant $\lambda_{S}$. Herein, by containing large amount of gas inside, our samples show increased $\lambda_{S}$, which suggests they can support better thermal conduction with relatively low density. The highest $\lambda_{S}$ of 0.75 (W/mk)/(g/cm^3^) was achieved for sample BN10, which is 82.9% higher than the virgin PE.

Previously, enormous theoretical works [42,43,44] have been conducted by investigators for establishing the thermal conduction mechanism framework. Prediction of the λ and its enhancement induced by fillers’ inclusion has been made by constructing the relationships between the λ and volume fraction of fillers. Herein, the apparent volume fraction of hBN is calculated and shown in Table 1. The $\lambda_{S}$ *vs* apparent volume fraction of hBN is presented in Figure 6B. From the figure, we can see the growth trend of $\lambda_{S}$ moderates along with the increase of hBN loading, which suggests an insignificant performance enhancement at high hBN loading. This phenomenon occurds due to the agglomeration of hBN in the PE matrix, which is a result of the poor interfacial adhesion between hBN and PE that leads to a bad dispersion of hBN in PE during the first melt compounding process [45].

In addition, for the purpose of comparing our PTCs with other reported bulk polyolefins (POs)/hBN blends that used different hBN types and hBN concentrations, we applied specific thermal conductivity enhancement ratio (TCE) *vs* filler’s volume fraction relationship for quantitative analysis. Herein, the TCE is given as:

$$TCE=100\times \left(\lambda_{C}/\rho_{C}-\lambda_{M}/\rho_{M}\right)/\left(\lambda_{M}/\rho_{M}\right)$$

where, $\lambda_{C}$ and $\lambda_{M}$ are the thermal conductivities of composite and matrix, respectively. $\rho_{C}$ and $\rho_{M}$ are the densities of composite and matrix, respectively. For our PTCs, the $\rho_{C}$ is calculated as shown in Section 2.3.2, while $\rho_{M}$ is directly measured. For the reported bulk composite, the $\rho_{C}$ is calculated as follows:

$$\rho_{C}=\left(1-V\right)\times \rho_{M}+V\times \rho_{F}$$

where V is the volume fraction of filler, and $\rho_{F}$ is the density of filler. For this comparison, the competitors are chosen to be bulk polyolefins filled with hBN [46,47,48,49], modified hBN [50,51,52] and boron nitride nanosheet (BNNS) [53,54,55,56], thus, the $\rho_{F}$ for calculation is selected to be 2.29 g/cm^3^ constantly.

**Figure 7:**
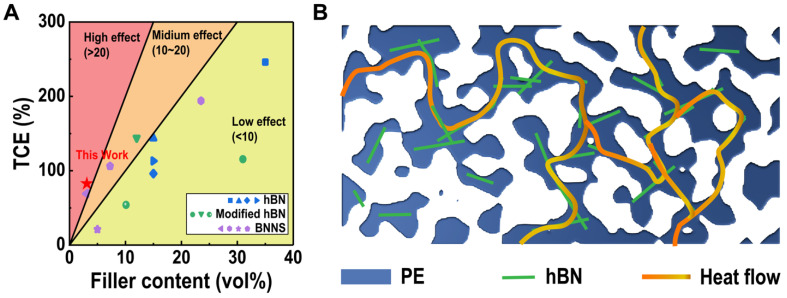
(**A**) The comparison between our PTCs and other reported bulk POs/hBN blends that filled with hBN, modified hBN and BNNS. (**B**) Illustration of the thermal conduction mechanism of our novel open-cell structural PTCs.

To analyze the effect of the filler on the TCE, TCE/V is used to represent the specific thermal conductivity enhancement ratio per unit volume fraction of filler inclusion. In this work, the value of TCE/V lower than 10 is defined as low effect. The value in range of 10–20 is regarded as medium effect, while value higher than 20 indicates high enhancement effect. From Figure 7A, it is clear that most hBN reinforced composites show low enhancement effect with TCE/V < 10. Such poor reinforcement effect is believed due to the immiscibility between the PO and hBN, which leads to bad agglomerations of hBN in PO matrix as high interfacial thermal resist. For the modified hBN included composites, their results are slightly better than hBN because of the improved compatibility between fillers and matrix. Among all three types of hBN, the POs/BNNS blends exhibit the highest TCE effect, which is mainly due to the less stack density of BNNS than either regular hBN and modified hBN, which benefits from the formation of thermal conduction pathway that can reduce interfacial thermal resist. For our PTC with 10 wt% hBN inclusion, the TCE/V value equals to 26.8, which is the largest value in this comparison. It is worth noting that these competitors are bulk samples with bare gas inside, while our sample still shows the best enhancement effect even though it contains approximately ~50 vol% ultralow-λ gas inside. Such comparison result indicates that our close-cellular PTCs can process higher specific thermal conductivities better than the bulk thermal conductive polymeric composites, and also suggests the advancement of our technique for fabricating lightweight thermal conductor.

To further indicates the thermal conduction mechanism of our advanced PTCs, we prepared an illustration as shown in Figure 7B. As revealed by the structural evaluation section, we have proved that our PTCs occupy large connectivity of pores. Thus, the remaining PE framework is also interconnected. As a consequence, the hBN layers inside PE are highly interconnected. As we mentioned in the introduction section, the thermal conduction mechanism of polymer/hBN composite is denominated by the phonon transportation. Therefore, the highly interconnected hBNs in our PTCs can serve as pathways for the phonon transfer, resulting into a superior reinforcement in thermal conductivity.

## 4. Conclusions

In this work, for the purpose of understanding how cellular architecture impacts the performance of polymer/fillers composites and in order to develop advanced porous thermal conductors, we fabricated porous PE/hBN composites through melt compounding plus salt leaching technique. The structural information was obtained by XRD and pore analysis. The XRD results indicate high alignments of hBN layers in the porous materials caused by compression molding. The pore structure analysis shows that the residual salt reduces both porosities and connectivities of the samples, while hBN inclusions can promote the interconnection of the salt that leads to higher porosities and connectivities. As a result, the porous PE/hBN in this work show enhanced thermal conductivities than the virgin PE. A comparison has been made between our open-celluar samples with the other bulk POs/hBN blends that reveals the best reinforcement effect on thermal conductivity in our work. In summary, we fabricated an advanced PTC with a high specific thermal conductivity of 0.75 (W/mk)/(g/cm^3^), which is 82.9% higher than the virgin PE, with a low hBN loading of 3.1 vol%. Furthermore, we believe this work can inspire the community of PTCs and draw their attention to the field of open-cellular PTCs for achieving better thermal conductivity enhancement effect on industrial applications.

## Figures and Tables

**Table 1:** Sample code, composition and calculated $V_{BN}$ of each sample.

Sample Code	Composition (wt%)			Apparent Volume Fraction of hBN in PTCs (vol%)	Apparent Density (g/cm^3^)
	PE	hBN	Salt		
5–50	47.5	2.5	50	1.5	0.71 ± 0.01
10–50	45	5	50	3.1	0.75 ± 0.02
15–50	42.5	7.5	50	4.7	0.78 ± 0.01

## Supplementary Materials

The following supporting information can be downloaded at: https://www.mdpi.com/article/10.3390/polym14050852/s1. Figure S1. SEM image of pristine hBN used in this work, Figure S2. Il-lustration of hot disk measurement, Figure S3. Photographs of each sample, Table S1. XRD peak intensities of each sample.
