# Supplementary material for: Lightweight Polyethylene/Hexagonal Boron Nitride Hybrid Thermal Conductor Fabricated by Melt Compounding Plus Salt Leaching
Source: Polymers (Basel). 2022 Feb 22;14(5):852. doi: 10.3390/polym14050852 (PMC8912592; doi:10.3390/polym14050852)
Supplement: Supplementary file 1 [file polymers-14-00852-s001.zip › polymers-1575753-supplementary.pdf]

Porous Polyethylene/Hexagonal Boron Nitride Hybrid Thermal Conductor Fabricated by Melt Compounding plus Salt Leaching

He-Jie Pi[1, 2, 3], Xiao-Xiao Liu[4, *], Jian-Yu Liao[1, 2, 3,*], Yue-Yun Zhou[1, 2, 3], Cong Meng[1, 2, 3]

[1] College of Urban and Environment Sciences，Hunan University of Technology, 88 Taishan Road, Zhuzhou City, Hunan Province,412007,China

[2] Hunan Provincial Key Laboratory of Comprehensive Utilization of Agricultural and Animal Husbandry Waste Resources，Hunan University of Technology, 88 Taishan Road, Zhuzhou City, Hunan Province,412007,China

[3] Hunan Key Laboratory of Water Safety Discharge in Urban and Its Resource Utilization，Hunan University of Technology, 88 Taishan Road, Zhuzhou City, Hunan Province,412007,China

[4] School of Advanced Manufacturing Technology, Guangdong Mechanical & Electrical Polytechnic, Guangzhou, China, 510550

* Corresponding to Xiao-Xiao Liu: 2020010016@gdmec.edu.cn

* Corresponding to Jian-Yu Liao: jyliao85@163.com

Supporting Information

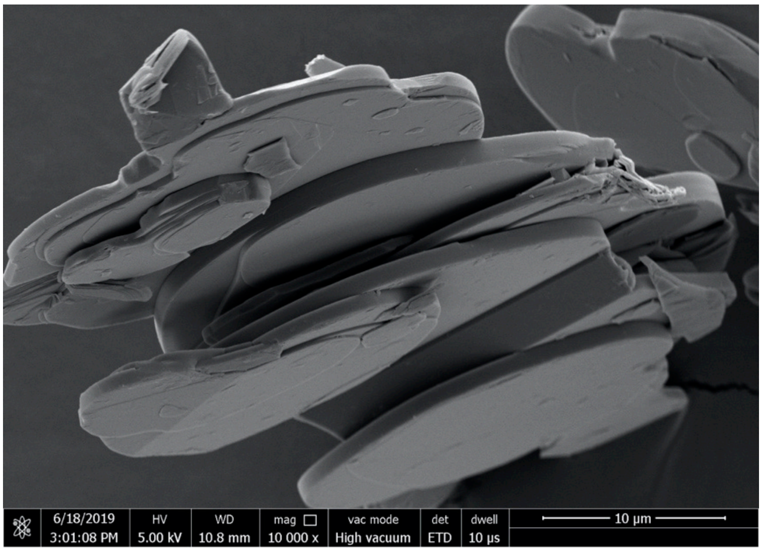

Figure S1. SEM image of pristine hBN used in this work.

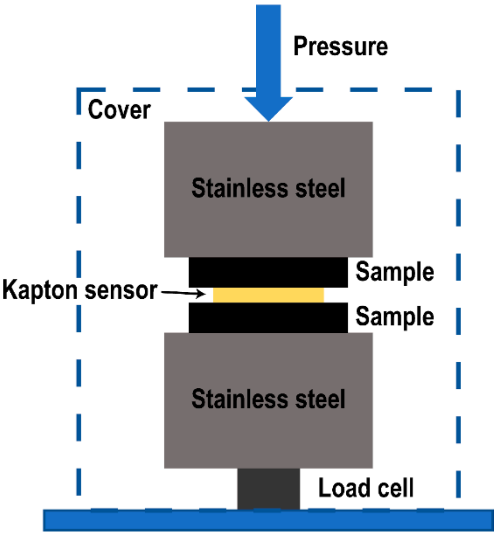

Figure S2. Illustration of hot disk measurement.

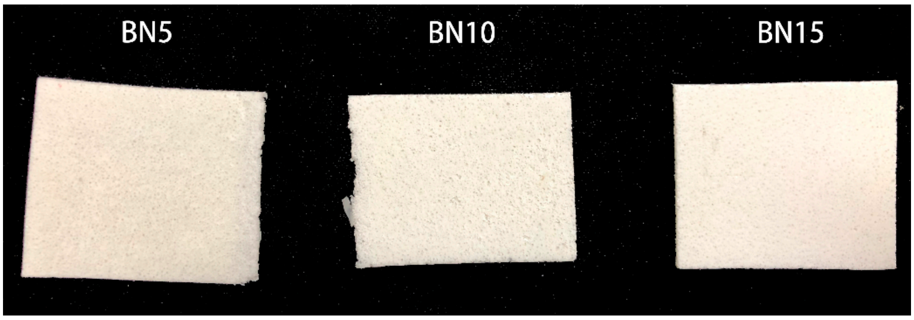

Figure S3. Photographs of each sample.

Table S1. XRD peak intensities of each sample.

SAMPLE	INTENSITY (A.U.)					
	**PE**		**HBN**			
	(110)	(020)	(002)	(100)	(004)	(103)
**PE**	36028.1	10145.9	/	/	/	/
**BN5**	25370	3384.8	12619.1	562	264	702.4
**BN10**	25712.4	3531.5	17625.4	594.9	283.5	751.7
**BN15**	25764.2	3673.9	21624	746	349.9	1007.3
**HBN**	/	/	138090.5	2536	1612.5	4977.6
